# A population-based survey of the epidemiology of symptom-defined gastroesophageal reflux disease: the Systematic Investigation of Gastrointestinal Diseases in China

**DOI:** 10.1186/1471-230X-10-94

**Published:** 2010-08-15

**Authors:** Jia He, Xiuqiang Ma, Yanfang Zhao, Rui Wang, Xiaoyan Yan, Hong Yan, Ping Yin, Xiaoping Kang, Jiqian Fang, Yuantao Hao, Qiang Li, John Dent, Joseph JY Sung, Duowu Zou, Mari-Ann Wallander, Saga Johansson, Wenbin Liu, Zhaoshen Li

**Affiliations:** 1 Department of Health Statistics, Second Military Medical University, Shanghai, China; 2 Department of Health Statistics, Xi'an Jiao Tong University, Xi'an, China; 3 Department of Health Statistics, Huazhong Science and Technology University, Wuhan, China; 4 Department of Health Statistics, Peking University, Beijing, China; 5 Department of Health Statistics, Zhongshan Medical University, Guangzhou, China; 6 Department of Gastroenterology, Hepatology & General Medicine, Royal Adelaide Hospital, Adelaide, SA, Australia; 7 Department of Medicine and Therapeutics, Chinese University of Hong Kong, Hong Kong, China; 8 Department of Gastroenterology, Changhai Hospital, Second Military Medical University, Shanghai, China; 9 AstraZeneca R&D Mölndal, Sweden; 10 Department of Public Health and Caring Sciences, Uppsala University, Sweden; 11 Previous Address: R&D Medical Affairs, AstraZeneca Pharmaceutical Company Limited, Shanghai, China; 12 Previous Address: AstraZeneca R&D Mölndal, Sweden

## Abstract

**Background:**

The epidemiology of gastroesophageal reflux disease (GERD) has yet to be investigated using the symptomatic threshold criteria recommended by the Montreal Definition. This study aimed to determine the prevalence of symptom-defined GERD across five regions of China, and to investigate variables associated with GERD.

**Methods:**

A representative sample of 18 000 adults (aged 18-80 years) were selected equally from rural and urban areas in each region (n = 1800). According to the Montreal Definition, GERD is present when mild symptoms of heartburn and/or regurgitation occur on ≥2 days a week, or moderate-to-severe symptoms of heartburn and/or regurgitation occur on ≥1 day a week.

**Results:**

In total, 16 091 participants completed the survey (response rate: 89.4%) and 16 078 responses were suitable for analysis. Applying the Montreal criteria, the prevalence of symptom-defined GERD was 3.1% and varied significantly (*p *< 0.001) among the five regions (from 1.7% in Guangzhou to 5.1% in Wuhan) and between rural and urban populations (3.8% vs 2.4%). Factors significantly associated with GERD included living in a rural area and a family history of gastrointestinal diseases.

**Conclusions:**

This population-based survey found that the prevalence of symptom-defined GERD in China was 3.1%, which is lower than that found in Western countries.

## Background

Gastroesophageal reflux disease (GERD) is a chronic disease that is associated with a range of troublesome symptoms, which can in turn have a significant impact on health-related quality of life and work productivity [1-4]. It is also associated with esophageal complications such as reflux esophagitis and Barrett's esophagus [1].

Interest in the epidemiology of GERD has grown during the past few decades, but interpretation of epidemiological studies of GERD has often been hampered by the use of inconsistent symptom-based definitions of the disease [5]. In 2005, Dent and colleagues performed a systematic review of studies that defined GERD as symptoms of heartburn and/or regurgitation occurring on at least 1 day per week [5]. They concluded that the prevalence of GERD was 10-20% in Western countries and approximately 5% in Asia based on this definition.

Since then, a global evidence-based consensus (the Montreal Definition of GERD) has recommended that in population-based surveys GERD should be defined as symptoms of heartburn and/or regurgitation that are either mild and occur on at least 2 days a week, or moderate-to-severe and occur on at least 1 day a week [1]. This was considered to be the level at which these characteristic GERD symptoms become troublesome. The epidemiology of symptom-defined GERD has yet to be investigated using these threshold criteria.

We have previously validated a survey methodology for the epidemiological study of GERD in Shanghai, China [6-8]. Here, we report results from the Systematic Investigation of Gastrointestinal Diseases in China (SILC), which is a large epidemiological survey of five regions of China [9]. The aim of the SILC study was to use the symptom threshold recommended for epidemiological studies by the Montreal Definition to determine the prevalence of symptom-defined GERD across five regions of China, and to investigate variables associated with this disease.

## Methods

### Setting, sampling and study design

The major population centres of Shanghai, Beijing, Xi'an, Wuhan and Guangzhou (including the rural districts surrounding the cities) were selected for sampling in this study. The demographic characteristics of these areas are summarized in Table 1. Fieldwork was carried out from April 2007 to January 2008.

**Table 1:** Demographic characteristics of the population centres included in the study.

	Shanghai^a^ (N = 13.5 m) %	Beijing^b^ (N = 11.8 m) %	Wuhan^b^ (N = 7.8 m) %	Xi'an^b^ (N = 7.3 m) %	Guangzhou^b^ (N = 7.3 m) %
**Female**	50.6	50.0	49.7	50.0	45.4
**Urban**	54.2	57.5	58.6	50.4	55.7
**Age (years)**					
18-29	13.3	25.6	31.1	27.8	31.9
30-39	18.8	21.5	19.6	25.7	28.5
40-49	30.2	22.0	20.3	19.9	17.8
50-59	16.4	15.1	15.5	12.9	11.5
60-69	12.4	9.3	8.0	8.8	6.3
70-80	9.1	6.5	5.5	5.0	4.0

^a^Data from the fifth population census in China (2000) [43,44].

^b^Data from the government 1% sample survey (2005) [45-47].

m, million.

As previously described [9], 18 000 residents of China aged 18-80 years were selected randomly using a stratified, multi-stage sampling methodology. Urban and rural populations, which have distinct socioeconomic characteristics (Additional File 1), were sampled in a ratio of 1:1 (n = 1800 from each stratum in each region) in proportion to the overall age and sex distribution of each region.

All respondents completed a survey consisting of a general information questionnaire and a Chinese version of the Reflux Disease Questionnaire (RDQ) [7]. A random sub-sample of 20% of the respondents in each region was asked to undergo a physical examination that included measurement of weight, height, and waist and hip circumference. The residents of Shanghai were also invited to undergo endoscopy, the results of which are described elsewhere [10,11].

The general information questionnaire collected self-reported information on age, height, weight, sex, marital status, education, income, occupation, lifestyle habits, health status, family history of gastrointestinal diseases, and medical history (current and previous medical problems and related treatment).

The RDQ was used to determine the frequency and severity of heartburn (defined as 'burning behind the breastbone' and/or 'pain behind the breastbone') and regurgitation (defined as 'acid taste in the mouth' and/or 'unpleasant movement of materials upwards from the stomach') during a 1-month recall period. The frequency and severity of each RDQ item are scored on a 6-point Likert scale (0-5; where 0 is no symptoms, 1 is symptoms on less than 1 day a week or very mild symptoms, and 5 is daily or severe symptoms) (Additional File 2). The validity and reliability of the RDQ as a diagnostic tool has been previously demonstrated [12]. The Chinese version of the RDQ used in the present study underwent extensive linguistic validation including forward and backward translation, cognitive debriefing of patients with GERD, and expert input from gastroenterologists. The pilot study showed that a version with a 1-week recall period had credible reliability and construct validity [7]. Applying the Montreal Definition, symptom-defined GERD was defined as mild symptoms of heartburn and/or regurgitation occurring on at least 2 days a week (a frequency score ≥3 and a severity score of ≥2 for any of the relevant symptoms), or moderate-to-severe symptoms of heartburn and/or regurgitation occurring on at least 1 day a week (a frequency score ≥2 and a severity score ≥3 for any of the relevant symptoms).

Participants filled out the questionnaires themselves, either in local residential committee offices or in their own home, with trained and supervised facilitators available to explain any questions that were unclear. Informed consent was obtained, and individuals were free to discontinue their participation in the study at any time. The study was approved by the Ethics Committee of the Second Military Medical University, Shanghai, China.

### Data collection and analysis

Data were collected and validated as previously described, with the SAS 9.1.3 program (SAS Institute, Cary, NC, USA) used to complete data analyses [9]. Odds ratios (ORs) and 95% confidence intervals (CIs) were calculated by univariate and multivariate logistic regression in order to examine factors that are potentially associated with symptom-defined GERD. The Cochran-Armitage test was used for trend testing and the Cochran-Mantel-Haenszel test was used to compare the baseline characteristics of respondents in the different study centres.

## Results

### Response rate and sample characteristics

In total, 16 091 individuals completed the survey (a response rate of 89.4%) and 16 078 responses (99.9%) were suitable for analysis. In the 20% sub-sample, the response rate was 89.4% and 3214 responses were suitable for analysis (99.8%). The mean (SD) age of participants in the total study sample was 42.5 (15.2) years; 52.2% of participants were female. Body mass index (BMI) ranged from 11.8 kg/m^2 ^to 41.0 kg/m^2^, with a mean (SD) of 22.6 kg/m^2 ^(3.3). The majority of participants reported that they did not drink alcohol (79.7%) or smoke cigarettes (69.9%). The baseline characteristics of each study centre are detailed in Additional File 3. Approximately half of all respondents lived in the rural areas of each of the studied provinces.

### Prevalence of reflux symptoms

At least monthly symptoms of heartburn and/or regurgitation were reported by 12.7% of participants (Table 2), with regurgitation (10.8%) being more prevalent than heartburn (4.0%). The prevalence estimates of individual RDQ items were as follows: 8.7% for 'an acid taste in the mouth', 5.3% for 'an unpleasant movement of material upwards from the stomach', 2.4% for 'burning behind the breastbone', and 2.8% for 'pain behind the breastbone'. Approximately 5% of participants experienced reflux symptoms on at least 1 day per week (Table 3). Regurgitation (4.2%) remained more common than heartburn in these participants (1.8%).

**Table 2:** Prevalence of at least monthly symptoms of heartburn or regurgitation (any frequency or severity).

Symptom	Prevalence (%)
**Heartburn or regurgitation**	**12.7**
Regurgitation	10.8
Acid taste in the mouth	8.7
Unpleasant movement of material upwards from the stomach	5.3
Heartburn	4.0
Burning behind the breastbone	2.4
Pain behind the breastbone	2.8

**Table 3:** Regional variation in the prevalence of reflux symptoms and symptom-defined gastroesophageal reflux disease (GERD).

Population	Reflux symptoms at least monthly n (%)	Reflux symptoms at least weekly n (%)	Symptom-defined GERD^a^ n (%)
Shanghai (n = 3151)	338 (10.7)	143 (4.5)	84 (2.7)
Beijing (n = 3168)	287 (9.1)	120 (3.8)	69 (2.2)
Wuhan (n = 3283)	532 (16.2)	245 (7.5)	169 (5.1)
Xi'an (n = 3266)	609 (18.6)	218 (6.7)	121 (3.7)
Guangzhou (n = 3210)	271 (8.4)	103 (3.2)	53 (1.7)
**Total (n = 16 078)**	**2037 (12.7)**	**829 (5.2)**	**496 (3.1)**

^a^Defined as mild symptoms of heartburn and/or regurgitation occurring on at least 2 days a week (Reflux Disease Questionnaire [RDQ] item frequency score ≥3 for a severity score of ≥2), or moderate-to-severe symptoms of heartburn and/or regurgitation occurring on at least 1 day a week (RDQ item frequency score ≥2 for a severity score ≥3).

**Figure 1:**
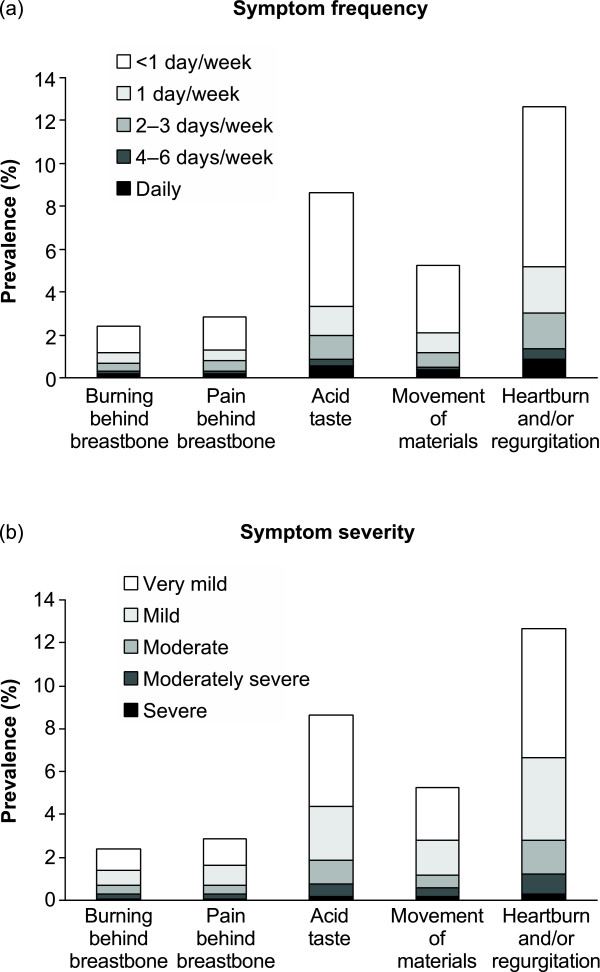
**Prevalence of reflux symptoms by (a) frequency and (b) severity (n = 16 078)**.

Most reflux symptoms were experienced less often than 1 day a week, and most were very mild in severity (Figures 1a and 1b).

### Prevalence of GERD

The prevalence of symptom-defined GERD was 3.1% overall (2.4% in urban areas and 3.8% in rural areas), and varied among the five regions studied, from 1.7% in Guangzhou to 5.1% in Wuhan (Table 3). The prevalence of symptom-defined GERD in the 20% sub-sample was 3.4%.

A total of 326 participants (2.0%) reported that they had previously been diagnosed with GERD by a physician: 73 of these met the study criteria for symptom-defined GERD and 253 did not (Table 4). The majority of the individuals who reported a history of GERD had been prescribed drug therapy at some point in time (76.1%; 248/326). The mean (SD) reported age for a first physician diagnosis of GERD was 37.8 (14.1) years. For almost all participants (99.7%), this physician diagnosis was more than 5 years ago.

**Table 4:** Self-reported history of GERD and treatment in participants with and without symptom-defined GERD.

Medical history of GERD	With symptom-defined GERD^a^ (n = 496) n (%)	Without symptom-defined GERD^a^ (n = 15 582) n (%)
**None **	423 (85.3)	15 328 (98.4)
**Previously diagnosed**	73 (14.7)	253 (1.6)
Treated^b^	57 (11.5)	191 (1.2)
Not treated	16 (3.2)	60 (0.4)

GERD, gastroesophageal reflux disease; RDQ, Reflux Disease Questionnaire.

^a^Defined as mild symptoms of heartburn and/or regurgitation occurring on at least 2 days a week (RDQ item frequency score ≥3 for a severity score of ≥2), or moderate-to-severe symptoms of heartburn and/or regurgitation occurring on at least 1 day a week (RDQ item frequency score ≥2 for a severity score ≥3).

^b^Drug treatment (excluding traditional Chinese medicine).

NOTE: numbers may not add up exactly where individual participants have refused to answer specific questions on the general information questionnaire.

### Factors associated with the presence of GERD

Factors found to be associated with the presence of symptom-defined GERD in a multiple logistic regression analysis are presented in Table 5. Participants living in rural areas were more likely to have symptom-defined GERD than those living in urban areas, whereas individuals aged 18-29 years were less likely to have symptom-defined GERD than those aged 30-39 years. Symptom-defined GERD was also associated with decreasing self-reported health status and a family history of gastrointestinal disease, and was inversely associated with recreational exercise taken at least once a week. Compared with participants without GERD, those with symptom-defined GERD were more likely to consume alcohol and were less likely to have attained a secondary/high school educational level. In univariate analysis, a BMI of ≥27.5 kg/m^2 ^was associated with a significant increase in the risk of GERD. Trend testing also showed a relationship between BMI and the occurrence of GERD (*p *= 0.003). However, there was no significant association between a BMI of ≥27.5 kg/m^2 ^and GERD in the multivariate analysis. Sex, smoking, occupation and income were not significantly associated with symptom-defined GERD.

**Table 5:** Characteristics of participants with and without symptom-defined GERD, and their association with symptom-defined GERD.

	With symptom-defined GERD^a^ (n = 496) n (%)	Without symptom-defined GERD^a ^(n = 15 582) n (%)	Univariate OR (95% CI)	Multivariate OR^b^ (95% CI)
**Environment**				
Urban	192 (38.7)	7880 (50.6)	1.00	1.00
Rural	304 (61.3)	7702 (49.4)	1.62 (1.35-1.95)	1.40 (1.13-1.72)
**Sex**				
Female	282 (56.9)	8108 (52.0)	1.00	1.00
Male	214 (43.1)	7474 (48.0)	0.82 (0.69-0.99)	1.00 (0.77-1.29)
**Age (years)**				
18-29	48 (9.7)	3632 (23.3)	0.49 (0.34-0.69)	0.67 (0.46-0.97)
30-39	97 (19.6)	3578 (23.0)	1.00	1.00
40-49	129 (26.0)	3683 (23.6)	1.29 (0.99-1.69)	1.11 (0.84-1.47)
50-59	113 (22.8)	2355 (15.1)	1.77 (1.34-2.33)	1.20 (0.89-1.62)
60-69	59 (11.9)	1444 (9.3)	1.51 (1.08-2.09)	0.82 (0.57-1.18)
70-80	50 (10.1)	890 (5.7)	2.07 (1.46-2.94)	0.85 (0.57-1.27)
**BMI (kg/m^2^)^c^**				
<18.5	44 (8.9)	1436 (9.2)	1.10 (0.79-1.53)	1.04 (0.73-1.48)
18.5-22.9	209 (42.1)	7512 (48.2)	1.00	1.00
23.0-27.4	181 (36.5)	5326 (34.2)	1.22 (1.00-1.50)	1.14 (0.92-1.41)
≥27.5	58 (11.7)	1244 (8.0)	1.68 (1.25-2.26)	1.32 (0.97-1.80)
**Education**				
None/primary school	183 (36.9)	2999 (19.2)	1.00	1.00
Secondary/high school	255 (51.4)	9675 (62.1)	0.43 (0.36-0.52)	0.62 (0.49-0.79)
College graduates or beyond	58 (11.7)	2906 (18.6)	0.33 (0.24-0.44)	0.69 (0.45-1.05)
**Occupation **				
Office worker	96 (19.4)	4116 (26.4)	1.00	1.00
Manual worker	400 (80.6)	11 445 (73.4)	1.50 (1.20-1.88)	0.88 (0.67-1.17)
**Total monthly family income (yuan)^d ^**				
≤1999	325 (65.5)	8490 (54.5)	1.00	1.00
2000-4999	139 (28.0)	5824 (37.4)	0.62 (0.51-0.76)	0.83 (0.67-1.04)
≥5000	31 (6.3)	1228 (7.9)	0.66 (0.45-0.96)	0.92 (0.61-1.39)
**Smoking status**				
Never smoker	355 (71.6)	10 875 (69.8)	1.00	1.00
Current smoker	122 (24.6)	4309 (27.7)	0.87 (0.70-1.07)	0.80 (0.60-1.06)
Ex-smoker	19 (3.8)	395 (2.5)	1.47 (0.92-2.36)	0.95 (0.56-1.61)
**Alcohol consumption^e^**				
No	393 (79.2)	12 420 (79.7)	1.00	1.00
Yes	103 (20.8)	3159 (20.3)	1.03 (0.83-1.28)	1.31 (1.00-1.71)
**Frequency of recreational exercise **				
Daily	350 (70.6)	10 009 (64.2)	1.00	1.00
At least weekly but less than daily	45 (9.1)	2149 (13.8)	0.60 (0.44-0.82)	0.68 (0.49-0.94)
Less than weekly	33 (6.7)	1330 (8.5)	0.71 (0.49-1.02)	0.87 (0.60-1.27)
Never	65 (13.1)	2066 (13.3)	0.90 (0.69-1.18)	0.81 (0.61-1.07)
**Self-reported health status **				
Very good	11 (2.2)	1759 (11.3)	1.00	1.00
Good	110 (22.2)	7471 (47.9)	2.35 (1.26-4.39)	2.33 (1.21-4.47)
Moderate	245 (49.4)	5558 (35.7)	7.05 (3.84-12.93)	6.43 (3.39-12.22)
Poor	121 (24.4)	741 (4.8)	26.11 (14.00-48.69)	20.10 (10.33-39.13)
Very poor	8 (1.6)	50 (0.3)	25.59 (9.86-66.37)	22.47 (8.34-60.52)
**Family history of GI diseases **				
No	392 (79.0)	14 252 (91.5)	1.00	1.00
Yes	104 (21.0)	1323 (8.5)	2.86 (2.29-3.57)	2.59 (2.05-3.28)

BMI, body mass index; CI, confidence interval; GERD, gastroesophageal reflux disease; GI, gastrointestinal; OR, odds ratio; RDQ, Reflux Disease Questionnaire.

^a^Defined as mild symptoms of heartburn and/or regurgitation occurring on at least 2 days a week (RDQ item frequency score ≥3 for a severity score of ≥2), or moderate-to-severe symptoms of heartburn and/or regurgitation occurring on at least 1 day a week (RDQ item frequency score ≥2 for a severity score ≥3).

^b^Adjusted by all variables in the table.

^c^BMI ranges are appropriate for the Asian population (underweight: < 18.5 kg/m^2^; normal: 18.5-22.9 kg/m^2^; overweight: 23.0-27.4 kg/m^2^; obese: ≥27.5 kg/m^2^) [48].

^d^Average yearly income in China in 2007: 24 932 yuan (~$3300) [42].

^e^Defined as alcohol consumed on at least four occasions per month.

NOTE: all data were generated from the general information questionnaire; numbers may not add up exactly where individual participants have refused to answer specific questions.

When separated into individuals who reported heartburn of any frequency or severity (n = 638) and those who reported regurgitation of any frequency or severity (n = 1738), a number of differences were found in the factors associated with regurgitation and heartburn. Alcohol consumption was significantly associated with the presence of regurgitation (OR: 1.34; 95% CI: 1.15-1.55) but not heartburn (OR: 1.15; 95% CI: 0.91-1.45). Conversely, a BMI of ≥27.5 kg/m^2 ^was significantly associated with the presence of heartburn (OR: 1.47; 95% CI: 1.14-1.91) but not regurgitation (OR: 1.10; 95% CI: 0.92-1.33). Compared with participants aged 30-39 years, those aged 70-80 years had a significantly higher risk of heartburn (OR: 1.45; 95% CI: 1.02-2.07) but a significantly lower risk of regurgitation (OR: 0.70; 95% CI: 0.55-0.90). Living in a rural area, decreasing health status and a family history of gastrointestinal disease were associated with a significant increase in the risk of both heartburn and regurgitation (data not shown).

No significant associations (in univariate or multivariate analyses) were found between symptom-defined GERD and BMI or waist-to-hip ratio among the 20% sub-sample who were randomly selected to undergo a physical examination (Table 6).

**Table 6:** Association of symptom-defined GERD with BMI and waist-to-hip ratio in participants who underwent a physical examination (20% sub-sample, n = 3214).

	With symptom-defined GERD^a^ (n = 110) n (%)	Without symptom-defined GERD^a^ (n = 3104) n (%)	Univariate OR (95% CI)	Multivariate OR^b^ (95% CI)
**BMI (kg/m^2^)^c^**				
<18.5	7 (6.4)	270 (8.7)	0.83 (0.37-1.85)	1.01 (0.45-2.29)
18.5-22.9	47 (42.7)	1504 (48.5)	1.00	1.00
23.0-27.4	41 (37.3)	1042 (33.6)	1.26 (0.82-1.93)	1.07 (0.68-1.68)
≥27.5	15 (13.6)	287 (9.2)	1.67 (0.92-3.03)	1.27 (0.65-2.46)
**Waist-to-hip ratio**				
Men: <0.90; women: <0.83	56 (50.9)	1801 (58.0)	0.86 (0.55-1.35)	1.10 (0.68-1.79)
Men: 0.90-0.95; women: 0.83-0.90	31 (28.2)	859 (27.7)	1.00	1.00
Men: >0.95; women: >0.90	23 (20.9)	414 (13.3)	1.54 (0.89-2.67)	1.42 (0.79-2.53)

BMI, body mass index; CI, confidence interval; GERD, gastroesophageal reflux disease; OR, odds ratio; RDQ, Reflux Disease Questionnaire.

^a^Defined as mild symptoms of heartburn and/or regurgitation occurring on at least 2 days a week (RDQ item frequency score ≥3 for a severity score of ≥2), or moderate-to-severe symptoms of heartburn and/or regurgitation occurring on at least 1 day a week (RDQ item frequency score ≥2 for a severity score ≥3).

^b^Adjusted by age, sex and all variables in the table.

^c^BMI ranges are appropriate for the Asian population (underweight: < 18.5 kg/m^2^; normal: 18.5-22.9 kg/m^2^; overweight: 23.0-27.4 kg/m^2^; obese: ≥27.5 kg/m^2^) [48].

NOTE: BMI data were missing for 1 participant, and waist-to-hip ratio data were missing for 30 participants.

The presence of symptom-defined GERD was associated with a self-reported history of dyspepsia (OR: 2.81; 95% CI: 2.20-3.59), dysphagia (OR: 4.56; 95% CI: 2.30-9.04), gastritis (OR: 3.06; 95% CI: 2.46-3.81) or peptic ulcer disease (OR: 2.05; 95% CI: 1.52-2.76). There was no significant association between GERD and self-reported irritable bowel syndrome (OR: 0.53; 95% CI: 0.12-2.42). Self-reported joint disorders (OR: 2.03; 95% CI: 1.58-2.61) and chronic cough (OR: 2.14; 95% CI: 1.39-3.28) were associated with GERD, whereas asthma (OR: 0.87; 95% CI: 0.39-1.94), hoarseness (OR: 0.76; 95% CI: 0.38-1.55) and non-cardiac chest pain (OR: 1.54; 95% CI: 0.72-3.31) were not.

## Discussion

This large multicentre study of the epidemiology of symptom-defined GERD surveyed a total population of 18 000 individuals from five regions across China. To our knowledge, it is the first epidemiological study anywhere in the world to apply the symptom-based criteria recommended by the Montreal Definition of GERD for use in population-based studies [1]. The overall prevalence of symptom-defined GERD in the present study was 3.1%. The prevalence varied widely between the regions studied, from 1.7% in Guangzhou to 5.1% in Wuhan, which emphasizes the cultural and demographic variability in this vast country and suggests that future epidemiological studies should not extrapolate findings in one area to the country as a whole.

Overall, 2% of study participants reported a pre-existing GERD diagnosis, and 76.1% of these individuals had received drug treatment for GERD, although the details of this treatment (e.g. drug name, dose and period of administration) were not recorded. Of those participants who had been diagnosed and treated for GERD, 77.0% did not reach the symptom threshold criteria for GERD used in this study, perhaps because their treatment was successful in controlling their symptoms. Conversely, 85.3% of participants who met symptom-defined GERD criteria had not been previously diagnosed with GERD. This suggests that consultation for reflux symptoms in this population is low, despite the negative impact that the symptoms are known to have on health-related quality of life [13].

The prevalence of symptom-defined GERD reported in the current study was lower than that found in the pilot study in Shanghai (6.2%) [6]. This is likely to be because the pilot study used a simple frequency threshold of reflux symptoms on at least 1 day a week to define GERD whereas the present study, in accordance with the Montreal Definition, excluded very mild symptoms of any frequency and mild symptoms on only 1 day a week from the definition of GERD.

The prevalence of GERD was comparable to or lower than that found in previous Chinese population-based surveys that used a variety of definitions of GERD (2.4-17.0%) [6,14-20], again reflecting the conservative definition used in the SILC study. The prevalence of symptoms of heartburn and/or regurgitation on at least 1 day a week was 5.2% in the SILC study, compared with 2.5-12.9% in previous studies conducted in China [6,14-20].

Despite the variation in the prevalence of GERD across the study centres, our results add weight to the conclusion that there is a lower prevalence of symptom-defined GERD in China (below 5%) than in Western countries (10-20%) [5]. This is mostly a result of a lower prevalence of heartburn (1.9-4.1% vs 7.7-17.8%) rather than regurgitation (5.5-7.8% vs 6.3-14.5%) in China [15,16] compared with Western populations [21]. Heartburn has also been found to be less common than regurgitation in Iran [22] and Turkey [23]. The reason for these differences in symptom patterns is unclear. They could be a result of genetic or pathophysiological differences between Western and Asian populations, although the evidence for this is limited [21]. The term heartburn is also less well understood in China than in Western countries. However, the linguistic validation performed as part of the current study [9] should have minimized the effect of such cultural differences.

### Factors associated with GERD

The factors most strongly associated with symptom-defined GERD were declining self-reported health status and a family history of gastrointestinal disease. Self-reported health status is known to be an accurate predictor of morbidity and mortality in China and other Asian populations as well as worldwide [24,25], and the association we observed may reflect the burden of symptoms and comorbidity in individuals with GERD. An association of GERD with a relevant family history has been seen in previous studies [5,26], and the results of twin studies in Sweden and the UK have also provided evidence of a genetic component to the disease [27-29]. In support of this genetic association, a recent study found an association between GERD and the gene encoding collagen type III alpha 1 (*COL3A1*) [30].

We also found that individuals living in a rural area had a higher risk of GERD than those living in an urban area. The reasons for this are unclear, and previous studies in China do not provide consistent support for this association. Pan *et al*. found that GERD was more common in rural than urban areas in Beijing (12.5% vs 8.6%) but that the reverse was true in Shanghai (7.0% vs 8.6%) [20]. Wang *et al*. found that GERD was more common in urban (21.1%) than rural (17.4%) areas of Xi'an [16].

The current study found a significant association between obesity and heartburn, but not between obesity and symptom-defined GERD; however, the pilot study using a less stringent definition of GERD did find a positive association with obesity [6]. Previous population-based studies that looked at the association of GERD with BMI in China have also reported inconsistent results [6,14-20]. This contrasts with the increasingly clear association between GERD and obesity that has been found in Western countries, particularly in the USA [31-34]. It is possible that this reflects differences in the prevalence of other etiological factors such as hiatus hernia or perhaps the low prevalence of obesity in China.

### Association of GERD with self-reported medical history

The current results support an association of GERD with a history of peptic ulcer disease [16,35-37], but do not support associations seen in Europe between GERD and irritable bowel syndrome or asthma [36,38,39] or a protective effect for gastritis against GERD [40]. It is, however, important to distinguish between objectively demonstrated gastritis and the patient-reported history of gastritis that is reported in the present study. In Asian countries 'gastritis' commonly denotes upper gastrointestinal discomfort and a diagnosis is unlikely to be based on endoscopic biopsy or serum pepsinogen measurement. GERD was associated with chronic cough in our study, supporting the established association with this extraesophageal syndrome [1].

The association we found between joint disorders (rheumatoid arthritis and osteoarthritis) and GERD may reflect the use of non-steroidal anti-inflammatory drugs (NSAIDs) for pain relief in this population [41].

### Strengths and limitations

This large, population-based, epidemiological study provides high-quality data on the prevalence of GERD in five geographically diverse regions of China. Key strengths are the large sample size, the use of a validated symptom questionnaire and survey methodology, and also the use of a symptom-based definition of GERD that was built on global consensus. Importantly, the population sampling and survey administration methods achieved a high response rate (89.4%) that minimized the potential for responder bias and generated representative adult population samples [9]. In addition, by gathering data on treatment history, it was possible to gain insight into how existing treatment of GERD may affect prevalence data.

Samples sizes were equal for rural and urban populations and for each population region, and were not weighted in proportion to the actual population size. However, available data indicate that the mean ratio of urban to rural people for all five study regions was approximately 0.81:1, ranging from 0.71:1 (in Wuhan) to 0.98:1 (in Beijing) [9] and in China overall this ratio was approximately 0.82:1 in 2007 [42]. Therefore we consider the urban and rural strata in this study to be sufficiently representative of the five study regions.

Language barriers and cultural differences are inevitable study limitations, although every effort was made to overcome these through the survey administration techniques and the linguistic validation of questionnaires [9]. Another potential limitation is that the medical history of participants was self-reported, although it should be noted that it is standard practice in China for patients to keep their own medical records which may reduce the impact of this limitation.

### Further study

Further investigation into potential risk factors and comorbidities associated with GERD is warranted, ideally in relation to incident cases of GERD. Ascertaining details of the drug treatments used by participants for diagnosed conditions would be of value in future studies, particularly use of NSAIDs, acetylsalicylic acid, traditional herbal remedies and treatments for GERD. Further research is needed to clarify whether the prevalence of GERD is increasing in China, and the factors that may be associated with such an increase.

## Conclusions

This study suggests that the prevalence of symptom-defined GERD is lower in China than in Western countries. This is mostly a result of a lower prevalence of heartburn rather than regurgitation in China compared with Western populations. The prevalence of symptom-defined GERD varies widely across China, which argues that future studies should not extrapolate findings in one area to the country as a whole.

## Competing interests

X. Yan, R. Wang, Y. Zhao, X. Ma, D. Zou, Z. Li, H. Yan, P. Yin, X. Kang, J. Fang, Y. Hao, and Q. Li declare that they have no competing interests. J. He has served as the Director of the Department of Health Statistics, Second Military Medical University and has received research funding from AstraZeneca. J. Dent has served as a speaker, a consultant and an advisory board member for AstraZeneca, and has received research funding from AstraZeneca. J.J.Y. Sung has served as a speaker, a consultant and an advisory board member for AstraZeneca, and has received research funding from AstraZeneca. S. Johansson is an employee of AstraZeneca. W. Liu and M-A. Wallander were employees of AstraZeneca at the time the study was conducted. W. Liu is now employed by Genzyme and M-A. Wallander is now employed by Bayer Healthcare. The study was funded by AstraZeneca R&D, Mölndal, Sweden. AstraZeneca had no role to play in the content and conduct of the study.

## Authors' contributions

JH, XM, YZ, RW, XY, JD, JJYS, DZ, M-AW, SJ, WL and ZL made substantial contributions to the conception and design of the study. JH, XM, YZ, RW, XY, HY, PY, XK, JF, YH, QL, WL and ZL participated in data collection. JH, XM, YZ, RW, XY, JD, JJYS, DZ, M-AW, SJ and ZL analyzed and interpreted the data. All authors have been involved in critically revising the manuscript for intellectual content, and have given final approval of the version to be published.

## Pre-publication history

The pre-publication history for this paper can be accessed here:

http://www.biomedcentral.com/1471-230X/10/94/prepub

## Supplementary Material

Additional file 1 **Baseline characteristics of respondents in urban and rural regions**. Click here for file

Additional file 2 **Scoring system of the Reflux Disease Questionnaire (RDQ)**. Click here for file

Additional file 3 **Baseline characteristics of respondents in each study centre, and the results of Cochran-Mantel-Haenszel trend testing comparing the prevalence of these characteristics across the study centres**. Click here for file
