# Supplementary material for: A population-based survey of the epidemiology of symptom-defined gastroesophageal reflux disease: the Systematic Investigation of Gastrointestinal Diseases in China
Source: BMC Gastroenterol. 2010 Aug 15;10:94. doi: 10.1186/1471-230X-10-94 (PMC2933714; doi:10.1186/1471-230X-10-94)
Supplement: Additional file 1 — Baseline characteristics of respondents in urban and rural regions. [file 1471-230X-10-94-S1.DOC]

**Additional File 1.** Baseline characteristics of respondents in urban and rural regions.

|  | **Urban region**  **N = 8072**  **(n [%])** | **Rural region**  **N = 8006**  **(n [ %])** |
| --- | --- | --- |
| **Sex** |  |  |
| Female | 4221 (52.3) | 4169 (52.1) |
| Male | 3851 (47.7) | 3837 (47.9) |
| **Age (years)** |  |  |
| 18−29 | 2060 (25.5) | 1620 (20.2) |
| 30−39 | 1756 (21.8) | 1919 (24.0) |
| 40−49 | 1838 (22.8) | 1974 (24.7) |
| 50−59 | 1239 (15.3) | 1229 (15.4) |
| 60−69 | 740 (9.2) | 763 (9.5) |
| 70−80 | 439 (5.4) | 501 (6.3) |
| **BMI (kg/m^2^)** |  |  |
| < 18.5 | 779 (9.7) | 701 (8.8) |
| 18.5–22.9 | 3901 (48.5) | 3820 (48.0) |
| 23.0–27.4 | 2763 (34.3) | 2744 (34.5) |
| ≥ 27.5 | 607 (7.5) | 695 (8.7) |
| **Education** |  |  |
| None/primary school | 836 (10.4) | 2346 (29.3) |
| Secondary/high school | 4781 (59.2) | 5149 (64.3) |
| College graduates or beyond | 2454 (30.4) | 510 (6.4) |
| **Occupation** |  |  |
| Office worker | 3077 (38.2) | 1135 (14.2) |
| Manual worker | 4984 (61.8) | 6861 (85.8) |
| **Total monthly family income (yuan)** | | |
| ≤ 1999 | 3348 (41.6) | 5467 (68.4) |
| 2000–4999 | 3734 (46.4) | 2229 (27.9) |
| ≥ 5000 | 965 (12.0) | 294 (3.7) |
| **Smoking status** |  |  |
| Never | 5857 (72.6) | 5373 (67.1) |
| Former | 214 (2.7) | 200 (2.5) |
| Current | 1998 (24.8) | 2433 (30.4) |
| **Alcohol consumption** |  |  |
| No | 6345 (78.6) | 6468 (80.8) |
| Yes | 1725 (21.4) | 1537 (19.2) |
| **Frequency of recreational exercise** | | |
| Daily | 4764 (59.1) | 5595 (70.0) |
| At least weekly but less than daily | 1312 (16.3) | 882 (11.0) |
| Less than weekly | 855 (10.6) | 508 (6.4) |
| Never | 1127 (14.0) | 1004 (12.6) |
| **Self-reported health status** |  |  |
| Very good | 783 (9.7) | 987 (12.3) |
| Good | 3836 (47.5) | 3745 (46.8) |
| Moderate | 3085 (38.2) | 2718 (34.0) |
| Poor | 345 (4.3) | 517 (6.5) |
| Very poor | 21 (0.3) | 37 (0.5) |
| **Family history of GI diseases** |  |  |
| No | 7298 (90.4) | 7346 (91.8) |
| Yes | 771 (9.6) | 656 (8.2) |

BMI, body mass index; GI, gastrointestinal.
