# Supplementary material for: A population-based survey of the epidemiology of symptom-defined gastroesophageal reflux disease: the Systematic Investigation of Gastrointestinal Diseases in China
Source: BMC Gastroenterol. 2010 Aug 15;10:94. doi: 10.1186/1471-230X-10-94 (PMC2933714; doi:10.1186/1471-230X-10-94)
Supplement: Additional file 2 — Scoring system of the Reflux Disease Questionnaire (RDQ). [file 1471-230X-10-94-S2.DOC]

**Additional File 2.** Scoring system of the Reflux Disease Questionnaire (RDQ).

| **Item** | **Frequency** | | | | | |
| --- | --- | --- | --- | --- | --- | --- |
|  | **None** | **Less than one day a week** | **One day**  **a week** | **2–3 days**  **a week** | **4–6 days a week** | **Daily** |
| Burning behind the breastbone | 0 | 1 | 2 | 3 | 4 | 5 |
| Pain behind the breastbone | 0 | 1 | 2 | 3 | 4 | 5 |
| Acid taste in the mouth | 0 | 1 | 2 | 3 | 4 | 5 |
| Unpleasant movement of material upwards from the stomach | 0 | 1 | 2 | 3 | 4 | 5 |
| Epigastric burning | 0 | 1 | 2 | 3 | 4 | 5 |
| Epigastric pain | 0 | 1 | 2 | 3 | 4 | 5 |
| **Item** | **Severity** | | | | | |
|  | **None** | **Very mild** | **Mild** | **Moderate** | **Moderately severe** | **Severe** |
| Burning behind the breastbone | 0 | 1 | 2 | 3 | 4 | 5 |
| Pain behind the breastbone | 0 | 1 | 2 | 3 | 4 | 5 |
| Acid taste in the mouth | 0 | 1 | 2 | 3 | 4 | 5 |
| Unpleasant movement of material upwards from the stomach | 0 | 1 | 2 | 3 | 4 | 5 |
| Epigastric burning | 0 | 1 | 2 | 3 | 4 | 5 |
| Epigastric pain | 0 | 1 | 2 | 3 | 4 | 5 |
