# Supplementary material for: A population-based survey of the epidemiology of symptom-defined gastroesophageal reflux disease: the Systematic Investigation of Gastrointestinal Diseases in China
Source: BMC Gastroenterol. 2010 Aug 15;10:94. doi: 10.1186/1471-230X-10-94 (PMC2933714; doi:10.1186/1471-230X-10-94)
Supplement: Additional file 3 — Baseline characteristics of respondents in each study centre, and the results of Cochran-Mantel-Haenszel trend testing comparing the prevalence of these characteristics across the study centres. [file 1471-230X-10-94-S3.DOC]

**Additional File 3**. Baseline characteristics of respondents in each study centre, and the results of Cochran–Mantel–Haenszel trend testing comparing the prevalence of these characteristics across the study centres.

|  | **Shanghai**  **N = 3151**  **(n [%])** | **Beijing**  **N = 3168**  **(n [%])** | **Wuhan**  **N = 3283**  **(n [%])** | **Xi’an**  **N = 3266**  **(n [%])** | **Guangzhou**  **N = 3210**  **(n [%])** | ***p***  **value** |
| --- | --- | --- | --- | --- | --- | --- |
| **Environment** |  |  |  |  |  |  |
| Urban | 1572 (49.9) | 1551 (49.0) | 1653 (50.4) | 1617 (49.5) | 1679 (52.3) | 0.077 |
| Rural | 1579 (50.1) | 1617 (51.0) | 1630 (49.6) | 1649 (50.5) | 1531 (47.7) | . |
| **Sex** |  |  |  |  |  |  |
| Female | 1749 (55.5) | 1682 (53.1) | 1719 (52.4) | 1647 (50.4) | 1593 (49.6) | < 0.001 |
| Male | 1402 (44.5) | 1486 (46.9) | 1564 (47.6) | 1619 (49.6) | 1617 (50.4) | . |
| **Age (years)** |  |  |  |  |  |  |
| 18−29 | 351 (11.1) | 758 (23.9) | 869 (26.5) | 811 (24.8) | 891 (27.8) | < 0.001 |
| 30−39 | 498 (15.8) | 669 (21.1) | 748 (22.8) | 824 (25.2) | 936 (29.2) | . |
| 40−49 | 1066 (33.8) | 730 (23.0) | 684 (20.8) | 692 (21.2) | 640 (19.9) | . |
| 50−59 | 594 (18.9) | 507 (16.0) | 506 (15.4) | 452 (13.8) | 409 (12.7) | . |
| 60−69 | 381 (12.1) | 311 (9.8) | 278 (8.5) | 310 (9.5) | 223 (6.9) | . |
| 70−80 | 261 (8.3) | 193 (6.1) | 198 (6.0) | 177 (5.4) | 111 (3.5) | . |
| **BMI (kg/m^2^)** |  |  |  |  |  |  |
| < 18.5 | 195 (6.2) | 190 (6.0) | 341 (10.5) | 366 (11.2) | 388 (12.1) | < 0.001 |
| 18.5–22.9 | 1359 (43.2) | 1208 (38.5) | 1704 (52.3) | 1670 (51.2) | 1780 (55.6) | . |
| 23.0–27.4 | 1267 (40.3) | 1322 (42.1) | 978 (30.0) | 1041 (31.9) | 899 (28.1) | . |
| ≥ 27.5 | 326 (10.4) | 421 (13.4) | 235 (7.2) | 184 (5.6) | 136 (4.2) | . |
| **Education** |  |  |  |  |  |  |
| None/primary school | 595 (18.9) | 427 (13.5) | 914 (27.8) | 659 (20.2) | 587 (18.3) | < 0.001 |
| Secondary/high school | 2162 (68.6) | 1884 (59.5) | 2056 (62.6) | 1920 (58.8) | 1908 (59.5) | . |
| College graduates or beyond | 394 (12.5) | 857 (27.1) | 312 (9.5) | 687 (21.0) | 714 (22.2) | . |
| **Occupation** |  |  |  |  |  |  |
| Office worker | 827 (26.3) | 797 (25.2) | 519 (15.8) | 1099 (33.7) | 970 (30.3) | < 0.001 |
| Manual worker | 2323 (73.7) | 2369 (74.8) | 2756 (84.2) | 2163 (66.3) | 2234 (69.7) | . |
| **Total monthly family income (yuan)** | | | | | | |
| ≤ 1999 | 1246 (39.6) | 1727 (54.7) | 2132 (65.0) | 2236 (68.5) | 1474 (46.2) | < 0.001 |
| 2000–4999 | 1574 (50.1) | 1240 (39.3) | 989 (30.1) | 899 (27.5) | 1261 (39.6) | . |
| ≥ 5000 | 324 (10.3) | 192 (6.1) | 160 (4.9) | 130 (4.0) | 453 (14.2) | . |
| **Smoking status** |  |  |  |  |  |  |
| Never smoker | 2144 (68.0) | 2125 (67.1) | 2294 (69.9) | 2272 (69.6) | 2395 (74.7) | < 0.001 |
| Ex-smoker | 81 (2.6) | 81 (2.6) | 90 (2.7) | 81 (2.5) | 81 (2.5) | . |
| Current smoker | 926 (29.4) | 962 (30.4) | 899 (27.4) | 912 (27.9) | 732 (22.8) | . |
| **Alcohol consumption** | | | | | | |
| No | 2523 (80.1) | 2388 (75.4) | 2490 (75.9) | 2738 (83.8) | 2674 (83.3) | < 0.001 |
| Yes | 628 (19.9) | 780 (24.6) | 791 (24.1) | 528 (16.2) | 535 (16.7) | . |
| **Frequency of recreational exercise** | | | | | | |
| Daily | 2097 (66.6) | 2151 (67.9) | 2436 (74.7) | 2063 (63.2) | 1612 (50.3) | < 0.001 |
| At least weekly but less than daily | 329 (10.4) | 318 (10.0) | 313 (9.6) | 579 (17.7) | 655 (20.4) | . |
| Less than weekly | 145 (4.6) | 191 (6.0) | 191 (5.9) | 355 (10.9) | 481 (15.0) | . |
| Never | 578 (18.4) | 507 (16.0) | 322 (9.9) | 267 (8.2) | 457 (14.3) | . |
| **Self-reported health status** | | | | | | |
| Very good | 164 (5.2) | 669 (21.1) | 402 (12.2) | 274 (8.4) | 261 (8.1) | < 0.001 |
| Good | 1347 (42.7) | 1417 (44.8) | 1517 (46.2) | 1525 (46.7) | 1775 (55.3) | . |
| Moderate | 1492 (47.4) | 983 (31.0) | 1029 (31.4) | 1255 (38.4) | 1044 (32.5) | . |
| Poor | 141 (4.5) | 87 (2.7) | 315 (9.6) | 201 (6.2) | 118 (3.7) | . |
| Very poor | 7 (0.2) | 10 (0.3) | 19 (0.6) | 11 (0.3) | 11 (0.3) | . |
| **Family history of GI disease** | | | | | | |
| No | 2664 (84.5) | 3065 (96.9) | 2886 (87.9) | 2991 (91.6) | 3038 (94.7) | < 0.001 |
| Yes | 487 (15.5) | 98 (3.1) | 396 (12.1) | 275 (8.4) | 171 (5.3) | . |

BMI, body mass index; GI, gastrointestinal.
